# Unraveling the Epigenetic Tapestry: Decoding the Impact of Epigenetic Modifications in Hidradenitis Suppurativa Pathogenesis

**DOI:** 10.3390/genes15010038

**Published:** 2023-12-26

**Authors:** Elena Maria Nardacchione, Paola Maura Tricarico, Ronald Moura, Adamo Pio d’Adamo, Ayshath Thasneem, Muhammad Suleman, Angelo Valerio Marzano, Sergio Crovella, Chiara Moltrasio

**Affiliations:** 1 Department of Advanced Diagnostics, Institute for Maternal and Child Health—IRCCS Burlo Garofolo, 34137 Trieste, Italy; elenamaria.nardacchione@burlo.trieste.it (E.M.N.); tricaricopa@gmail.com (P.M.T.); ronaldmoura1989@gmail.com (R.M.); adamopio.dadamo@burlo.trieste.it (A.P.d.); 2 Department of Medical Surgical and Health Sciences, University of Trieste, 34127 Trieste, Italy; 3 Laboratory of Animal Research Center (LARC), Qatar University, Doha 2713, Qatar; a.thasneem@qu.edu.qa (A.T.); m.suleman@qu.edu.qa (M.S.); sgrovella@qu.edu.qa (S.C.); 4 Biological Science Program, Department of Biological and Environmental Sciences, College of Arts and Sciences, Qatar University, Doha 2713, Qatar; 5 Dermatology Unit, Fondazione IRCCS Ca’ Granda Ospedale Maggiore Policlinico, 20122 Milan, Italy; angelo.marzano@unimi.it; 6 Department of Pathophysiology and Transplantation, Università degli Studi di Milano, 20122 Milan, Italy

**Keywords:** hidradenitis suppurativa, acne inversa, epigenetics, methylation, histone modifications, microRNAs, long non-coding-RNAs

## Abstract

Hidradenitis suppurativa (HS) is a chronic autoinflammatory skin disorder, which typically occurs during puberty or early adulthood. The pathogenesis of HS is complex and multifactorial; a close interaction between hormonal, genetic, epigenetics factors, host-specific aspects, and environmental influences contributes to the susceptibility, onset, severity, and clinical course of this disease, although the exact molecular mechanisms are still being explored. Epigenetics is currently emerging as an interesting field of investigation that could potentially shed light on the molecular intricacies underlying HS, but there is much still to uncover on the subject. The aim of this work is to provide an overview of the epigenetic landscape involved in HS. Specifically, in this in-depth review we provide a comprehensive overview of DNA methylation/hydroxymethylation, histone modifications, and non-coding RNAs (such as microRNA—miRNA-132, miRNA-200c, miRNA-30a-3p, miRNA-100-5b, miRNA-155-5p, miRNA-338-5p) dysregulation in HS patients. An interesting element of epigenetic regulation in HS is that the persistent inflammatory milieu observed in HS lesional skin could be exacerbated by an altered methylation profile and histone acetylation pattern associated with key inflammatory genes. Deepening our knowledge on the subject could enable the development of targeted epigenetic therapies to potentially restore normal gene expression patterns, and subsequentially ameliorate, or even reverse, the progression of the disease. By deciphering the epigenetic code governing HS, we strive to usher in a new era of personalized and effective interventions for this enigmatic dermatological condition.

## 1. Introduction

Hidradenitis suppurativa (HS) is a chronic-relapsing, debilitating autoinflammatory skin disorder of the pilosebaceous unit. It is clinically characterized by painful deep-seated nodules, abscesses, draining tracts, and hypertrophic scarring [1]. According to recent estimates, HS prevalence ranges from under 1% to 4% of the population [2] but these percentages are likely underestimated due to under-and/or misdiagnosis, which often leads to a significant diagnostic delay [3]. An average delay of 7–10 years has been reported between the first presentation of symptoms and a final HS diagnosis [4]. HS typically occurs during puberty or early adulthood, between 10 and 21 years of age [5]; moreover, novel findings suggest that HS has a bimodal age of onset, with the first peak in the late teens and a second peak in the mid-40s [6].

HS poses a significant medical challenge due to its complex pathogenesis. While clinical manifestations of HS are well-documented, the molecular intricacies that propel its onset and progression remain a subject of intense exploration. A strict interaction between hormonal, genetic, epigenetics factors, host-specific aspects, and environmental influences contribute to the susceptibility, onset, severity, and clinical course of this disease. Furthermore, an innate and adaptive immunity dysfunction leading to autoinflammation has been reported to play a pivotal role with upregulation of proinflammatory cytokines such as Interleukin (IL)-1β and IL-17, chemokines, and tumor necrosis factor (TNF)-α both in lesional skin and in serum of HS patients [1,7,8]. Although progress is being made, the exact molecular mechanisms underlying this disease, particularly the epigenetic ones, have not yet been clarified.

Epigenetics, a burgeoning field that investigates heritable and acquired modifications influencing gene function without altering the DNA sequence, is emerging as a promising avenue to decode the enigma of HS [9]. The human epigenome, encompassing DNA methylation, histone modifications, and non-coding RNAs, orchestrates gene expression dynamics, playing a pivotal role in governing various cellular processes. In the context of HS, a deeper exploration of the epigenetic landscape becomes essential to comprehend the factors contributing to disease development and devise targeted therapeutic strategies [10].

Variations in methylation patterns have been linked to several inflammatory skin disorders, including, among others, psoriasis, and atopic dermatitis (AD). In HS, too, the analysis of specific changes in DNA methylation patterns can unravel altered gene expression profiles, potentially unveiling novel therapeutic targets. Histone modifications, the wielders of chromatin structure and gene accessibility, form another pivotal layer of the epigenetic landscape. Investigating patterns of histone modifications in HS-affected tissues may shed light on the regulation of inflammatory pathways, providing insights into the chronic inflammation characteristic of this condition [11]. In the last decades, non-coding RNAs, comprising microRNAs (miRNAs) and long non-coding RNAs (lncRNAs), have emerged as key players in epigenetic regulation. These molecules exert intricate control over gene expression and may significantly contribute to the dysregulated immune responses observed in HS. Delving into the repertoire of dysregulated non-coding RNAs in HS can furnish a comprehensive understanding of the molecular underpinnings of the disease [12].

The distinctive anatomical locations affected by HS further underscore the importance of epigenetic regulation. Exploring site-specific epigenetic modifications in the unique microenvironments of intertriginous areas may elucidate factors influencing disease severity and clinical manifestations. Moreover, understanding the interplay between genetic predisposition and epigenetic modifications is of paramount importance. Unraveling how genetic factors and environmental triggers converge to shape the epigenetic landscape in HS offers a holistic perspective on disease etiology [7,13].

In this in-depth narrative review, we synthesize current knowledge regarding the epigenetic landscape in HS. Our goal is to provide a comprehensive overview of DNA methylation, histone modifications, and non-coding RNA dysregulation in HS patients. Additionally, we explore the potential interplay between genetic factors and epigenetic modifications, paving the way for future research directions in decoding the mysteries of HS pathophysiology. Embarking on this exploration of the intricate world of epigenetics in HS, our ultimate aspiration is to contribute to the development of targeted therapeutic strategies that address the root causes of this debilitating condition. By deciphering the epigenetic code governing HS, we strive to usher in a new era of personalized and effective interventions for this enigmatic dermatological disorder.

## 2. Search Strategy

### Criteria for Paper Selection

To provide an overview of the current state of knowledge regarding the topic of epigenetic landscape in HS, papers were selected from those included in the electronic databases PubMed/MEDLINE, Google Scholar, Scopus, and Web of Science over the past 20 years. The following search terms were used: hidradenitis suppurativa, acne inversa, keratinocytes, epidermal differentiation, epigenetics, epigenetic regulators, methylation, histones, microRNAs, long-noncoding RNAs, inflammatory skin diseases, environmental factors. Each selected paper was analyzed, the data extracted, and presented in the main text to provide an overview of the main aspects of epigenetic regulation that contributes to the HS pathogenesis.

## 3. Harmony of Change: Exploring Epigenetic Variations and Their Influence on Gene Expression Modulation

**Figure 1:**
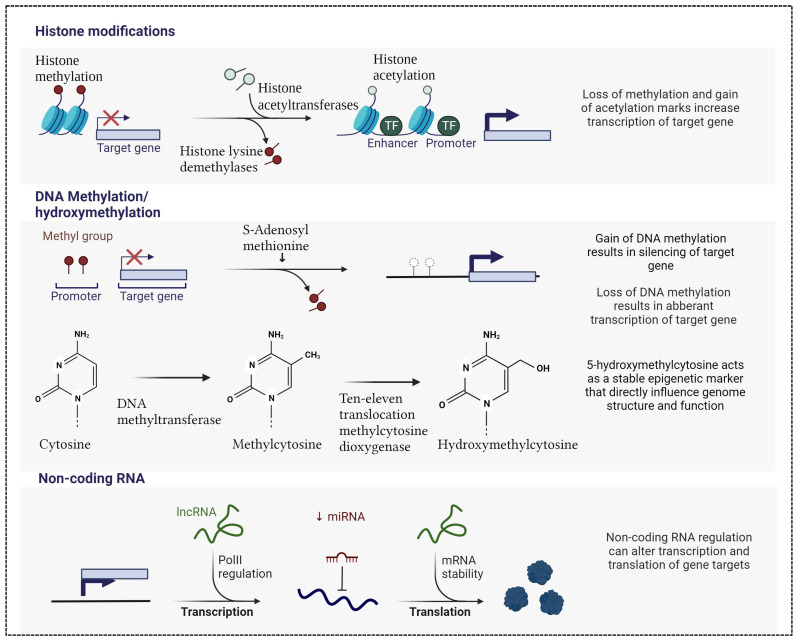
Main epigenetic marks include histone modifications in terms of histone methylation and acetylation, DNA methylation/demethylation and hydroxymethylation, non-coding RNA including long non-coding (lcn) and microRNA (miRNA). Created with Biorender.

Epigenetic changes, consisting of alterations in DNA methylation, histone modifications, and dysregulation of non-coding RNA activity, exert a profound influence on gene transcription and translation, and consequently on the intricate modulation of human diseases. These dynamic modifications play a pivotal role in regulating gene expression profiles without altering the DNA sequence (Figure 1).

DNA methylation, the most common epigenetic mark, involves the covalent addition of a methyl group from S-adenyl methionine (SAM) to the fifth carbon (C-5) position of a cytosine by DNA methyltransferases (DNMTs), forming a 5-methylcytosine (5-mC). DNA methylation regulates gene expression by recruiting proteins involved in gene repression or by inhibiting the binding of transcription factor(s) to DNA [14]. This mechanism is essential for regulating tissue-specific gene expression, silencing retroviral elements, genomic imprinting, and other several functions that contribute to cellular homeostasis [14]. In line with these essential roles, a growing number of human diseases have been found to be related with aberrant DNA methylation patterns [15].

Not only methylation, but also demethylation takes on a significant role in inflammatory and immunity cell signaling pathways by influencing the transcription of several proinflammatory mediators [16]. 5-hydroxymethylcytosine (5-hmC) is the hydroxylation product of 5-mC and is considered to be the first reaction leading to demethylation. This process is catalyzed by the ten-eleven translocation (Tet) family of enzymes, namely Tet1, Tet2, and Tet3, in combination with isocitrate dehydrogenases (IDH1, IDH2, and IDH3) [17]. The roles of Tet proteins in regulating chromatin architecture and gene transcription, independently of DNA methylation, has been gradually discovered, although their exact regulatory role remains unknown [16].

Histone modifications are another crucial aspect of the epigenetic landscape, which encompass alterations to the proteins around which DNA is wound, known as histones. These modifications impact chromatin structure, regulating its physical properties and thus influencing gene accessibility and transcriptional state [18]. Various histone modifications, including acetylation, methylation, and phosphorylation, intricately orchestrate the regulation of gene expression, thus playing key roles in most biological processes that are involved in DNA manipulation and expression. Dysregulation of histone modifications is implicated in a broad range of diseases, from cancer to neurodegenerative disorders, underscoring their significance in maintaining cellular homeostasis [19].

Non-coding RNAs, particularly miRNAs, represent a distinct class of epigenetic regulators that post-transcriptionally modulate gene expression. MiRNAs are small, single-stranded molecules containing 21 to 23 nucleotides that operate by binding to messenger RNAs (mRNAs) and inhibiting their translation or promoting their degradation [20]. They are powerful regulators of several cellular activities including cell growth, differentiation, development, and apoptosis [21]. Similar to the aforementioned epigenetic marks, miRNAs have been linked to many diseases; for example, in cardiovascular disorders, aberrant miRNA expression profiles contribute to pathological processes, including inflammation, apoptosis, and fibrosis [22]. MiRNAs are also abundant as extracellular circulating miRNAs (c-miRNAs) and have the great potential to be available as biomarkers in various diseases [23].

As mentioned above, the dysregulation of these epigenetic mechanisms contributes significantly to a spectrum of human disorders. In cancer, for instance, global changes in DNA methylation patterns and alterations in histone modifications often lead to the silencing of tumor suppressor genes or to the activation of oncogenes [24], while in cardiovascular diseases, abnormal epigenetic modifications influence key processes such as endothelial function, vascular remodeling, and cardiac hypertrophy [25].

Understanding the specific types and functions of epigenetic variations is crucial for unraveling the complex pathogenesis of human diseases. Ongoing research seeks to decipher the intricate interactions between epigenetic regulators and specific genes associated with various diseases. Such insights hold promise for the development of targeted epigenetic therapies, ushering in a new era of precision medicine. By selectively modulating these epigenetic mechanisms, it may be possible to restore normal gene expression patterns and, in turn, ameliorate or even reverse the progression of diseases. As the field of epigenetics continues to advance, it provides fertile ground for innovative therapeutic interventions and a deeper understanding of the molecular underpinnings of human health and disease [26].

Epigenetic modifications also play a crucial role in the epidermal development and keratinocyte differentiation program, orchestrating the intricate molecular events that lead to the formation and maintenance of the skin’s outermost layer. In this scenario, epigenetic regulators exhibit both activating and repressive effects on chromatin in keratinocytes via the adenosine triphosphate (ATP)-dependent chromatin remodelers, histone demethylases, and genome organizers that promote terminal keratinocyte differentiation, and the DNA methyltransferases, histone deacetylases, and Polycomb components that stimulate proliferation of progenitor cells and inhibit premature activation of terminal differentiation-associated genes [27]. During epidermal differentiation, DNA methylation significantly contributes to the regulation of gene expression. Indeed, specific genes associated with cell cycle regulation, keratinocyte differentiation, and barrier formation undergo dynamic changes in DNA methylation patterns, influencing their transcriptional activity. The methylation status of key genes, such as those encoding keratins and proteins involved in cell adhesion, is finely tuned to ensure proper epidermal development and function [28].

Histone modifications also play a pivotal role in epidermal differentiation by modulating the accessibility of chromatin and regulating gene expression [29]. Acetylation, which involves the addition of an acetyl group to the ε-amino group of lysine through histone acetyltransferases (HATs) enzymes-, and methylation of histones are among the critical modifications that occur during various stages of epidermal development, contributing to the formation of a chromatin landscape conducive to the activation or repression of specific genes involved in keratinocyte differentiation and skin barrier formation [30].

Likewise, histone deacetylation and demethylation play a crucial role in coordinating the crosstalk between signaling pathways with chromatin remodeling and gene expression regulators [31]. Histone deacetylases (HDACs) are a class of enzymes that remove acetyl groups from an ε-N-acetyl lysine amino acid on a histone, resulting in a more closed chromatin structure with repression of gene expression [32]. It has been demonstrated that HDACs 1/2 are particularly involved in hair follicle formation as well as epidermal development and stratification [33], whereas histone demethylases promote terminal keratinocyte differentiation [27]. Non-coding RNAs, particularly miRNAs, add an additional layer of complexity to the epigenetic regulation of epidermal differentiation, by participating in the fine-tuning of the differentiation process and helping to maintain the delicate balance between proliferation and differentiation in the epidermis [34].

The coordinated interplay of these epigenetic mechanisms ensures the precise execution of epidermal differentiation [35]. Aberrations in epigenetic regulation can lead to skin diseases characterized by impaired barrier function, abnormal keratinization, and compromised skin integrity. Understanding the epigenetic control of epidermal differentiation provides insights into the molecular basis of skin health and pathology, offering potential avenues for therapeutic interventions in dermatological conditions. As research in epigenetics advances, it opens new possibilities for targeted treatments aimed at restoring or modulating epigenetic marks to promote healthy epidermal development and function.

## 4. Unraveling the Impact of Epigenetic Landscape in Hidradenitis Suppurativa

### 4.1. The Role of DNA Methylation

**Figure 2:**
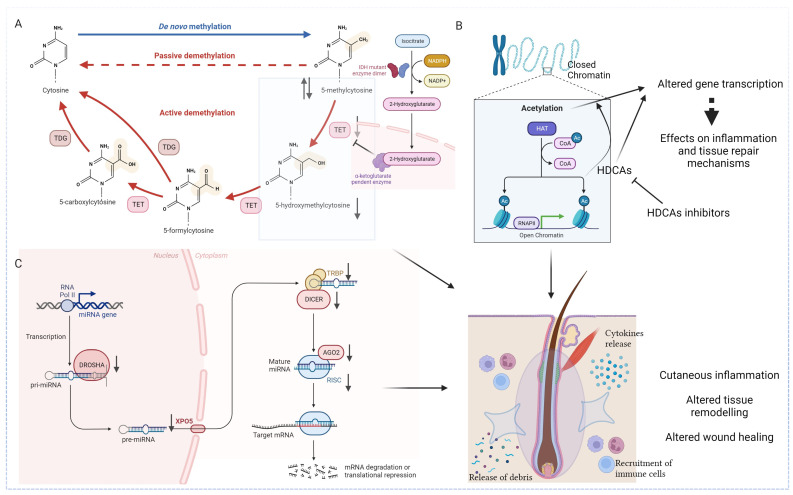
Epigenetic landscape in HS. (**A**) Studies investigating the epigenetic landscape in HS revealed specific alterations in DNA methylation profiles (hyper and hypomethylation). DNA hydroxymethylation, with the formation of 5-hydroxymethylcytosine also plays a causative role. The under expression of the latter, partly due to a significant reduction of Tet and IDH enzymes, leads to an unbalanced global methylation pattern. (**B**) The dysregulation of histone acetylation patterns have been reported in dysregulated immune responses, chronic inflammation and defective wound healing. Epigenetic modulators that influence histone acetylation, such as HDACs inhibitors, are now being studied in various inflammatory and autoimmune disorders. (**C**) Biogenesis and mechanism of action of miRNAs in HS patients is characterized by pronounced dysregulation of the principal regulators of miRNAs, leading to an abnormal inflammatory response, aberrant keratinocyte proliferation, and impaired wound healing. IDH: isocitrate dehydrogenase (NADP(+)) 1; TET: ten-eleven translocation methylcytosine dioxygenase; TDG: thymine-DNA glycosylase; HAT: histone acetyltransferases; CoA: coenzyme A; Ac: acetylation; HDAC: histone deacetylase; RNAPII: RNA polymerase II; miRNA: microRNA; XPO5: exportin-5; TRBP: transactivation response element RNA-binding protein; AGO2: argonaute RISC Catalytic Component 2; RISC: RNA-induced silencing complex; ⇅ over-expressed and under expressed, respectively. Created with Biorender.

Studies investigating the epigenetic landscape in HS revealed specific alterations in DNA methylation profiles (Figure 2A), mainly involving the modulation of inflammatory pathways. An abnormal increase in DNA methylation, also called hypermethylation, of genes related to anti-inflammatory cytokines and immune response modulation may contribute to the uncontrolled and persistent inflammation characteristic of HS lesional skin. Dysregulated methylation patterns in genes associated with wound healing and tissue repair could also impact the resolution of lesions and contribute to the chronicity of this disease. Additionally, the silencing of genes involved in the regulation of apoptotic processes may contribute to the persistence of inflammatory infiltrates in affected skin regions [36].

Concerning immune dysregulation, the Janus kinase/signal transducer and activator of transcription (JAK-STAT) signaling pathway has been observed to be aberrantly methylated in HS patients’ blood [11]. In normal conditions, interferon-γ (*IFNG*) and IFN-γ receptor 1 (*IFNGR1*), through their binding, activates JAK-STAT pathway, which in turn induces the upregulation of interferon-stimulated genes (ISGs), triggering antiviral and adaptive immune responses [37]. In a recent study conducted by Radhakrishna et al. [11], *IFNG* and *IFNGR1* genes were hypo- and hypermethylated, respectively, thus leading to an altered immune and inflammatory response.

Similarly, *MTOR* (Mechanistic Target Of Rapamycin Kinase) gene that acts as the target for the cell-cycle arrest and immunosuppressive effects of the FKBP12 (12-kDa FK506-binding protein)-rapamycin complex was found to be significantly hypermethylated in peripheral blood samples of HS patients [11]. The inhibition of mTOR with rapamycin inhibits T and B cells activation by reducing their sensitivity to IL-2, which exerts a fundamental immunoregulatory role [38]. A dysregulation in T and B cells signaling pathways play several impactful roles in skin inflammation and abnormal phenotypes. It has been demonstrated that mTOR dysregulation is involved in several inflammatory diseases, including HS, which could represent a molecular marker related to disease severity. Moreover, Balato et al. [39] analyzed, on HS patients, the effects of adalimumab, the only TNF-α antagonist approved by Food and Drug Administration (FDA) and EMA (European Medicines Agency) for moderate-to-severe HS, on mTORC1 (mTOR Complex 1) activity, highlighting both the specific involvement of mTORC1 in HS pathogenesis and its potential prominent role as possible new mechanism by which TNF-α inhibition improves HS.

In the above-mentioned study conducted by Radhakrishna et al. [11], several other hypo- and/or hypermethylated genes belonging to inflammation, immune responses, and keratinization pathways have been reported, thus sustaining that HS can be considered as an autoinflammatory keratinization disorder [40]. Moreover, these genes were enriched in the 117 different pathways, including IL-4/IL-13 pathways and Wnt/β-catenin signaling. The latter is a highly conserved mechanism that plays a prominent role in maintaining cellular homeostasis; it regulates embryonic development, cell proliferation and differentiation, apoptosis, and inflammation-associated cancer [41]. This finding also correlates with the fact that the risk of cancer among HS-affected patients is very high [42].

In another study conducted by Wang et al. [43], whole-genome DNA methylation sequencing on lesional and unaffected skin derived from HS patients was performed. The authors found an hypermethylation of CXC chemokine ligand 16 (CXCL16), which acts as a mediator of the innate immunity of epidermal keratinocytes by attracting CXC chemokine receptor (CXCR) 6-expressing cells, such as activated T cells and Natural Killer (NK) T cells [44]. Hypermethylation of CXCL16 induced decreased expression of this chemokine, which could affect chemotaxis of CXCR6-bearing cells thus impairing both innate and adaptive immune responses in HS patients. In addition, considering the antimicrobial properties of CXCL16, its downregulation could also promote bacterial colonization by perpetuating the inflammatory loop [43].

The impact of DNA methylation in HS extends beyond immune dysregulation and inflammation. Epigenetic alterations in genes related to skin structure and function, as well as those involved in the regulation of the hair follicle cycle, have also been implicated. This suggests that the epigenetic modulation of tissue-specific genes could contribute to the structural changes and to the recurrent nature of HS lesions [36]. Radhakrishna et al. [45] also investigated telomere-related genes (TRGs) methylomes in whole blood-derived genomic DNA of HS patients to assess their role in HS pathogenesis, reporting 585 differentially methylated sites in 585 TRGs (474 hypomethylated and 111 hypermethylated). TRGs act on multiple pathways such as DNA repair, telomere maintenance, mismatch repair, and cell cycle control, and their disruption led to the telomeres shortening, which is associated with HS progression, aging, cellular senescence, and an increased risk of cancer and associated comorbidities, such as metabolic syndrome, cardiovascular disease, and inflammatory disorders.

As anticipated, DNA hydroxymethylation seems to play a role in the epigenetic landscape of inflammatory skin diseases as well, and to contribute to HS pathogenesis, too. 5-hmC levels have been observed to be significantly reduced in HS patients’ lesional and perilesional tissues compared with those of healthy controls. This specific epigenetic mark appears to have a role in the inflammatory microenvironment, even though the mechanism by which this happens is yet to be investigated [28]. Interestingly, TeT and IDH gene expression in HS perilesional and lesional skin has been also investigated, revealing that abnormal expression of the DNA hydroxymethylation regulators may play a significant role in HS pathogenesis [15]. Hessam et al. [15] reported that mRNA of Tet1/2/3 and IDH1/2/3a/3b was significantly underexpressed in HS lesional regions when compared to healthy skin. The expression of IDH1 and IDH2 was also lower in HS perilesional skin, while Tet3 was significantly lower in HS lesional regions when compared to HS perilesional skin.

Although this topic requires further study and validation, these promising results suggest that molecular targets that restore the normal expression and/or activity of these important regulators could have a huge positive impact at the therapeutic level. Targeting specific DNA methylation patterns could in fact represent a novel promising approach for managing HS patients. Epigenetic therapies, including DNA demethylating agents, are being explored in various dermatological conditions and could hold promise for HS treatment by reversing aberrant methylation patterns and restoring normal gene expression.

The role of DNA methylation/hydroxymethylation in modulating inflammatory and tissue-specific immunity cell pathways suggests that epigenetic dysregulation contributes to the complex pathogenesis of HS. Unraveling the epigenetic signatures associated with HS would not only improve our understanding of the disease but also pave the way for the development of targeted therapies aimed at correcting specific methylation patterns and, consequently, alleviating the burden of this chronic debilitating autoinflammatory skin condition. This potential is represented not only by the exploration of DNA methylation, but also of non-coding RNAs and histone acetylation [46].

### 4.2. Investigating the Role of Histone Acetylation

Histone acetylation is emerging as a critical player in the complex pathophysiology of HS (Figure 2B). The dysregulation of histone acetylation patterns has been reported in dysregulated immune responses, chronic inflammation, and defective wound healing [10]. One of the central aspects of histone acetylation in HS primarily involves the modulation of inflammatory pathways. An altered histone acetylation pattern associated with key inflammatory genes may contribute to the persistent inflammatory milieu observed in HS lesions, while a dysregulated histone acetylation in the promoters of genes encoding for cytokines, chemokines, and immune cell activation mediators could perpetuate an environment conducive to the formation of painful nodules and abscesses [10].

Moreover, the role of histone acetylation extends to the dysregulation of genes involved in tissue repair and wound healing. Aberrant acetylation patterns in genes associated with extracellular matrix (ECM) remodeling, cell proliferation, and angiogenesis may impede the resolution of lesions, contributing to the chronicity of HS. The impaired balance between histone acetylation and deacetylation processes may disrupt the finely tuned orchestration of inflammatory responses and tissue repair mechanisms in affected skin regions [10].

From a therapeutic point of view, epigenetic modulators that influence histone acetylation, such as HDACs inhibitors, already approved by the US Food and Drug Administration (FDA) for the treatment of refractory cutaneous T-cell lymphoma [47], are now being studied in various inflammatory and autoimmune disorders, showing beneficial therapeutic effect in various murine models of these conditions [48,49,50,51]. In this context, pan-HDAC inhibitor vorinostat has been studied in primary keratinocyte (KC) culture obtained from psoriatic skin, revealing that it was able to inhibit KCs proliferation and induce their differentiation and apoptosis. Moreover, a reduced psoriasiform phenotype with a decreased epidermal thickness and inhibition of keratinocyte proliferation was demonstrated [52].

Although these results need to be further evaluated, especially in terms of safety, they offer a promising treatment option for both psoriasis and other hyperproliferative skin disorders, such as HS.

### 4.3. The Role of microRNAs and Long Non-Coding RNA

In recent years, the role of non-coding RNAs, specifically miRNAs and lncRNAs, has garnered attention in understanding the molecular intricacies of HS pathogenesis. As mentioned above, miRNAs are small non-coding RNAs of about 22 nucleotides that play a pivotal role in post-transcriptional gene regulation.

The role of miRNAs in the pathogenetic scenario of HS was supported by the research of Hessam et al. [53], demonstrating a significant decrease in the expression of principal regulators of miRNAs maturation in the affected skin of HS patients [54] (Figure 2C). The same authors reported that the biogenesis and mechanism of action of miRNAs in HS patients were characterized by pronounced dysregulation leading to an abnormal inflammatory response, aberrant keratinocyte proliferation, and impaired wound healing [45,54]. In detail, Hessam et al. [54] demonstrated that Drosha and Dicer, RNA processing enzymes required for the maturation of miRNAs, and the RNA-induced silencing complex (RISC) were downregulated in lesional HS patients’ skin samples. It is worth noting that Drosha and Drosha co-factor DGRC8 were downregulated in healthy-appearing perilesional skin of HS patients, suggesting their potential involvement in the subclinical inflammatory response. In contrast, the authors assumed that the downregulation of Dicer and exportin-5 (XPO5), whose function is to export pre-miRNAs out of the nucleus and into the cytoplasm for further processing by the Dicer enzyme, might contribute to the acute inflammatory phase when active HS lesions occurred [54].

The expression of all RISC components, namely trans-activation response (TAR) RNA binding protein 1 (TRBP1), TRBP2, Protein ACTivator of the interferon-induced protein kinase (PACT), Argonaute RISC Catalytic Component-1 (AGO1) and Component-2 (AGO2), metadherin, and staphylococcal nuclease and Tudor domain-containing-1 (SND1), were investigated, showing a significant decrease in HS lesional skin compared to healthy controls and thus demonstrating the causative role of miRNAs in HS pathogenesis [55].

Other miRNAs (Table 1), such as miRNA-24-1-5p, miRNA-26a-5p, miRNA-206, miRNA-338-3p, and miRNA-146a-5p, have been found to be reduced in peripheral blood leukocytes from HS patients [56]. In contrast, a reduced expression of miRNA-125b-5p has been observed in HS lesional skin compared to healthy-appearing perilesional skin [53]. Most of these miRNAs are involved in vascular homeostasis and ECM regulation, suggesting a potential link between their dysregulation and the vascular abnormalities and tissue remodeling seen in HS. Another example is miRNA-100-5b, whose expression appeared downregulated as a consequence of defective nicastrin (NCSTN) expression, which then led to the upregulation of p-AKT (also known as protein kinase B-PKB-) to promote keratinocytes proliferation [57].

Conversely, other miRNAs have been found to be overexpressed in HS lesional skin such as miRNA-21-5p, miRNA-31-5p, miRNA-146a-5p, miRNA-155-5p, and miRNA-223-5p. Of note, only miRNA-155-5p also showed an increased expression in perilesional skin compared to healthy controls [53]. In addition, an overexpression of miR-338-5p was found in lesional regions of HS patients [56]. Considering the properties of this miRNA, which are closely associated with HS invasiveness and production of pro-inflammatory mediators, it has been recently proposed as a non-invasive clinical biomarker for HS [56].

Interestingly, He et al. [58] investigated the relationship between *NCSTN* mutations and familial HS pathogenesis by studying differential miRNAs expression and their related pathways. The authors demonstrated that *NCSTN* pathogenic variants led to decreased miR-30a-3p levels, which in turn downregulated Ras-Related Protein Rab-31 (RAB31) expression. Moreover, high levels of this protein accelerated the degradation of activated epidermal growth factor receptor (EGFR), leading to aberrant keratinocyte differentiation. Based on these findings, the authors assumed that in HS familial cases *NCSTN* mutated, the *NCSTN*/miRNA-30a-3p/RAB31 axis contributes to impaired activation of the EGFR signaling pathway, followed by dysregulated keratinocyte differentiation.

Recently, Radhakrishna et al. [46], comparing genomic DNA extracted from HS and healthy control whole blood, identified miRNA gene methylation profiles, including 54 hypomethylated and 6 hypermethylated genes as potentially associated with HS. The majority of these miRNAs are responsible for skin development, keratinocyte proliferation, inflammation, and wound healing. In particular, miRNA-29, miRNA-200, miRNA-205, miRNA-548, and miRNA-132 play a crucial role in skin homeostasis; miR-200c acts as a crucial factor in regulating skin repair whereas miRNA-132 participates in wound repair and keratinocyte proliferation. It is evident that an alteration of methylation pattern of these miRNAs has a strong impact on HS pathogenesis.

In conclusion, understanding the specific miRNA signatures associated with different stages of HS and distinct clinical manifestations could provide valuable insights into disease progression and inform future targeted therapeutic strategies [56]. LncRNAs, a diverse class of RNA molecules exceeding 200 nucleotides, have also emerged as key players in HS pathophysiology. LncRNAs can regulate gene expression through various mechanisms, including chromatin remodeling, transcriptional regulation, and interaction with other cellular components [59]. In HS, dysregulated expression of lncRNAs, such as MALAT1 (Metastasis Associated Lung Adenocarcinoma Transcript 1) has been recently reported. These lncRNAs may influence pathways related to inflammation, tissue remodeling, and immune response, contributing to the chronicity of HS [11].

The intricate interplay between miRNAs and lncRNAs in HS extends beyond individual molecules, forming complex regulatory networks, as single lncRNAs may be associated with several miRNAs and mRNAs, and vice versa. For example, certain lncRNAs may serve as sponges for miRNAs, sequestering them and preventing their inhibitory effects on target mRNAs. Such competitive endogenous RNA networks could be crucial in maintaining the balance of gene expression in HS-affected skin regions.

The dysregulation of miRNAs and lncRNAs in HS adds a layer of complexity to our understanding of the disease. These non-coding RNAs play diverse roles in regulating inflammatory responses, tissue remodeling, and immune function, contributing to the chronic and recurrent nature of HS lesions. Modulating the expression or activity of specific miRNAs or lncRNAs could potentially correct the dysregulated gene expression patterns, offering a novel approach to treating HS. However, the complexity of these regulatory networks and the potential for off-target effects underscore the need for careful investigation and validation of specific non-coding RNA candidates as potential therapeutic targets [55].

## 5. Conclusions and Future Perspectives

HS stands as a complex and enigmatic dermatological disorder that presents a great challenge for dermatologists and researchers [60]. The quest to decipher the intricacies of this disease has witnessed remarkable strides with the advent of genomics, transcriptomics, and proteomics. These multiomic approaches have significantly enhanced our understanding of the molecular underpinnings of HS, shedding light on genetic risk factors, aberrant gene expression patterns, and the intricate protein networks orchestrating disease pathology.

**Figure 3:**
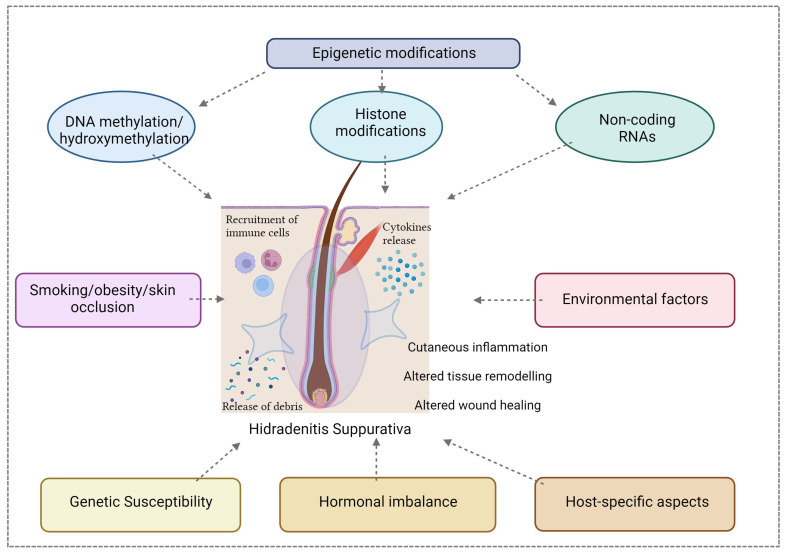
The pathogenesis of HS is complex and multifactorial; a close interaction between epigenetics factors (including DNA methylation, histone modifications and non-coding RNAs), hormonal, genetic, host-specific aspects and environmental influences contributes to the susceptibility, onset, severity, and clinical course of this disease. Created with Biorender.com.

The PlatOMICS platform has been instrumental in consolidating and harmonizing these diverse datasets, offering researchers a comprehensive resource for exploration [61]. Yet, to unlock the full spectrum of HS complexities, an imperative next step is the integration of epigenomic data. The epigenome, encompassing DNA methylation, histone modifications, and non-coding RNAs, introduces an additional layer of regulatory control, modulating gene expression dynamics without altering the DNA sequence. Inclusion of the epigenomic component is of paramount importance for unraveling the nuanced regulatory mechanisms governing HS pathogenesis (Figure 3).

Integrating genomics [62], transcriptomics [63,64], proteomics [65], and epigenomics [11] is pivotal for achieving a holistic understanding of HS [66]. Genomic studies have unveiled potential susceptibility genes and risk loci associated with disease-onset; transcriptomic analyses have provided insights into gene expression signatures while proteomics is helping to elucidate the complex protein landscapes. The synergy of these -omic layers can unveil the interconnected molecular pathways driving HS, providing a comprehensive map of the disease landscape. However, this integration necessitates the development of a user-friendly platform adept at seamlessly harmonizing and navigating the intricate relationships between genetic, transcriptomic, proteomic, and epigenomic factors in HS [67]. Such a platform would empower researchers to conduct in-depth analyses, identify key regulatory elements, and discern dysregulated pathways in the disease state. The democratization of access to this integrated platform would foster collaboration among researchers and clinicians, facilitating a collective effort to decipher HS complexities.

In line with a recent study conducted by Jin et al. [68], in which it has been demonstrated the epigenetic plasticity of epidermal progenitors cells in HS, it would be particularly useful to study the epigenetic profiles of tissues or cells involved in HS, as a more specific investigation may help elucidate factors involved in clinical manifestations and disease severity, and also would possibly lay the foundations to develop new therapeutic approaches. Furthermore, a collaborative approach is essential for accelerating discoveries, validating findings, and translating research insights into effective therapeutic strategies. By fostering a culture of knowledge-sharing and collaborative exploration, researchers can collectively contribute to a deeper understanding of HS, ultimately paving the way for innovative therapeutic interventions and personalized treatment strategies.

In conclusion, the journey to comprehend HS in its entirety requires a holistic approach that integrates genomics, transcriptomics, proteomics, and epigenomics- particularly at a tissue or cell specific level. The synergistic exploration of these multi-omic dimensions, facilitated by a user-friendly platform, holds tremendous promise for unraveling the intricate etiology and pathogenetic mechanisms underlying HS. This integrative effort not only advances our molecular understanding but also opens avenues for targeted therapeutic interventions and personalized treatment strategies, heralding a new era in the management of this complex dermatological condition.

## Figures and Tables

**Table 1:** The most common aberrant expressed miRNAs in HS.

Peripheral Blood Leukocytes		
miRNA	Effect	Reference
miRNA-24-1-5p 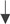	Inhibition of interferon gamma expression	[56]
miRNA-26a-5p 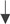	Effects on cell proliferation, apoptosis, migration and interferon gamma pathway	[56]
miRNA-206 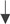	Effects on inflammation mediated by chemokine and cytokine signaling pathway; insulin/IGF pathway-mitogen activated protein kinase kinase/MAP kinase cascade; interferon gamma pathway	[56]
miRNA-338-3p 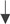	Dysfunction of regulatory T cells	[56]
miRNA-146a-5p 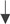	Effects on inflammation mediated by chemokine and cytokine signaling pathway; interferon gamma pathway	[56]
**HS lesional skin**		
miRNA-125b-5p 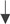	Inhibition of TNF-alpha, regulating the differentiation and proliferation of keratinocytes through its inhibitory effect on the FGFR2	[53]
miRNA-100-5b 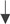	Effects on cellular self-renewal and wound healing	[57]
miRNA-30a-3p 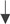	In the presence of *NCSTN* mutations and RAB31, aberrant keratinocytes differentiation	[58]
miRNA-146a-5p 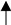	Effects on TRAF6/IRAK-1 and inhibition of TNF-alpha production	[53]
miRNA-21-5p 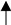	Effects on T cell-derived skin inflammation	[53]
miRNA-31-5p 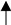	Overexpression of proinflammatory mediators in keratinocytes	[53]
miRNA-155-5p 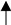	Effects on Th17 cells differentiation and function; keratinocyte proliferation and differentiation; apoptosis; overexpression of TNF-alpha	[53]
miRNA-223-5p 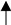	Effects on proliferation of progenitors and differentiation and activation of granulocytes	[53]
miRNA-338-5p 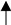	Stimulation of cell proliferation, invasion, and the production of cytokines IL-1a, IL-6, and COX2	[56]
**Whole blood**		
miRNA-200 family— altered methylation	Effects on wound healing and regeneration	[46]
miRNA-132 family— altered methylation	Upregulation during the inflammation phase of wound repair	[46]
miRNA-548 family— altered methylation	Effects on cellular viability, proliferation and migration	[46]

MiRNA: micro-RNA; IGF: insulin-like growth factor; TNF: tumour necrosis factor; FGFR2: fibroblast growth factor receptor 2; NCSTN: nicastrin; RAB31: Ras-Related Protein Rab-31; TRAF6: TNF Receptor Associated Factor 6; IRAK.1: Interleukin-1 receptor-associated kinase 1; Th17: T helper 17 cell; IL: interleukin; COX2: cyclooxygenase-2. ⇅ over-expressed and under expressed, respectively.

## Data Availability

Not applicable.
